# Antiviral activity and possible mode of action of ellagic acid identified in *Lagerstroemia speciosa* leaves toward human rhinoviruses

**DOI:** 10.1186/1472-6882-14-171

**Published:** 2014-05-26

**Authors:** Sang Wook Park, Min Jung Kwon, Ji Young Yoo, Hwa-Jung Choi, Young-Joon Ahn

**Affiliations:** 1 Interdisciplinary Program in Agricultural Biotechnology, College of Agriculture and Life Sciences, Seoul National University, Seoul 151-921, Republic of Korea; 2 Department of Agricultural Biotechnology, Seoul National University, Seoul 151-921, Republic of Korea; 3 Department of Infection Biology, Zoonosis Research Center, Wonkwang University School of Medicine, Iksan 570-749, Jeollabuk-do, Republic of Korea

**Keywords:** Human rhinovirus, Natural antiviral agent, *Lagerstroemia speciosa*, Ellagic acid, Tannin, Cytotoxicity, Selectivity, RNA replication

## Abstract

**Background:**

Human rhinoviruses (HRVs) are responsible for more than half of all cases of the common cold and cause billions of USD annually in medical visits and school and work absenteeism. An assessment was made of the cytotoxic and antiviral activities and possible mode of action of the tannin ellagic acid from the leaves of *Lagerstroemia speciosa* toward HeLa cells and three rhinoviruses, HRV-2, -3, and -4.

**Methods:**

The antiviral property and mechanism of action of ellagic acid were evaluated using a sulforhodamine B assay and real-time reverse transcription-PCR (RT-PCR) with SYBR Green dye. Results were compared with those of the currently used broad-spectrum antiviral agent, ribavirin.

**Results:**

As judged by 50% inhibitory concentration values, natural ellagic acid was 1.8, 2.3, and 2.2 times more toxic toward HRV-2 (38 μg/mL), HRV-3 (31 μg/mL), and HRV-4 (29 μg/mL) than ribavirin, respectively. The inhibition rate of preincubation with 50 μg/mL ellagic acid was 17%, whereas continuous presence of ellagic acid during infection led to a significant increase in the inhibition (70%). Treatment with 50 μg/mL ellagic acid considerably suppressed HRV-4 infection only when added just after the virus inoculation (0 h) (87% inhibition), but not before -1 h or after 1 h or later (<20% inhibition). These findings suggest that ellagic acid does not interact with the HRV-4 particles and may directly interact with the human cells in the early stage of HRV infections to protect the cells from the virus destruction. Furthermore, RT-PCR analysis revealed that 50 μg/mL ellagic acid strongly inhibited the RNA replication of HRV-4 in HeLa cells, suggesting that ellagic acid inhibits virus replication by targeting on cellular molecules, rather than virus molecules.

**Conclusions:**

Global efforts to reduce the level of antibiotics justify further studies on *L. speciosa* leaf-derived materials containing ellagic acid as potential anti-HRV products or a lead molecule for the prevention or treatment of HRV infection.

## Background

Human rhinoviruses (HRVs) (Picornaviridae) are the most frequent cause of mild upper respiratory tract infection, or common cold. They are responsible for more than half of all cases of the common cold [1,2]. HRVs are also associated with more severe diseases such as acute otitis media in children [3] and sinusitis in adults [4]. HRVs can also cause severe lower respiratory tract infections such as pneumonia [5], wheezing [6], bronchiolitis, and exacerbations of asthma and chronic obstructive pulmonary disease [2] in infants and children as well as fatal pneumonia in elderly and immunocompromised adults [7]. Although HRV-induced upper respiratory illness is often mild and self-limiting, the socioeconomic impact caused by medical visits and school and work absenteeism by HRV infection is considerable and the degree of inappropriate antibiotic use is significant [2,8,9]. More than 100 serotypes of HRV become an obstacle of development of a unifying vaccine [10]. There is, therefore, a high need for the development of selective antiviral agents with novel target sites to establish an effective HRV management strategy and tactics because there are currently no approved antiviral therapies for the prevention or treatment of HRV infection [2].

Plants have been suggested as alternative sources for antiviral products largely because they constitute a potential source of bioactive secondary substances that have been perceived by the general public as relatively safe, with minimal impacts to human health, and often act at multiple and novel target sites [11-14]. Much effort has been focused on plants and their constituents as potential sources of commercial antiviral products for prevention or treatment of HRV infection. In the screening of plants for anti-HRV activity, a methanol extract from the leaves of Giant Crape-myrtle (called banaba in the Phillippines), *Lagerstroemia speciosa* (L.) Pers. (Lythraceae), was shown to have good antiviral activity toward HRV-4. Very little information has been done to consider potential use of *L. speciosa* to manage HRV, although the plant leaves possess antidiabetic and hypoglycemic [15], antiobesity [16], antioxidant [17], antigout [18], antiinflammatory [19], and antibacterial activities [20].

The aim of the study was to assess the cytotoxic and antiviral effects on HeLa cells and three HRV serotypes (HRV-2, HRV-3, and HRV-4) of the tannin ellagic acid from *L. speciosa* leaves, compared to commercial pure ellagic acid and ribavirin, a currently used broad-spectrum antiviral agent [21]. The antiviral property and mechanisms of action of the constituent were elucidated using sulforhodamine B (SRB) assay and real-time reverse transcription-PCR with SYBR Green dye.

## Methods

### Instrumental analysis

^1^H and ^13^C NMR spectra were recorded in DMSO-*d*_6_ on a JNM-ECX 400 spectrometer (Jeol, Tokyo, Japan) at 400 and 100 MHz, respectively, using tetramethylsilane as an internal standard, and chemical shifts are given in δ (ppm). UV spectra were obtained in methanol on a BioMate 5 spectrophotometer (Thermo Spectronic, Rochester, NY), Fourier transform infrared (FT-IR) spectra on a Nicolet Magna 550 series II spectrometer (Midac, Atlanta, GA), and mass spectra on a Jeol GSX 400 spectrometer. Silica gel 60 (0.063–0.2 mm) (Merck, Darmstadt, Germany) was used for column chromatography. Merck precoated silica gel plates (Kieselgel 60 F_254_, 0.20 mm) were used for analytical thin-layer chromatography (TLC). A SCL-10 AVP high-performance liquid chromatograph (Shimadzu, Kyoto, Japan) was used for isolation of active principles.

## Materials

Commercially available pure ellagic acid (≥95% purity) and SRB were purchased from Sigma-Aldrich (St. Louis, MO). The antiviral agent ribavirin was supplied by Tokyo Chemical Industry (Tokyo). Anitbiotic-antimycotic and minimum essential medium (MEM) were purchased from Invitrogen (Grand Island, NY). Fetal bovine serum was supplied by PAA Laboratories (Etobicoke, Ontario, Canada). All of the other chemicals and reagents used in this study were of analytical grade quality and available commercially.

### Human rhinovirus serotypes and cell line

HeLa (ATCC CCL-2), a human epithelial adenocarcinoma cervix cell line, was purchased from the American Type Culture Collection (ATCC) (Manassas, VA). The cell line was maintained in MEM supplemented with 10% fetal bovine serum and 0.01% antibiotic-antimycotic in a humidified incubator at 37°C and 5% CO_2_. HRV-2 (ATCC VR-1112AS/GP), HRV-3 (ATCC VR-1113), and HRV-4 (ATCC VR-1114AS/GP) were purchased from ATCC. The three HRVs were propagated in HeLa cells at 37°C. Virus titers were determined by cytopathic effects (CPE) in HeLa cells and were expressed as 50% cell culture infective dose (CCID_50_) per mL as described previously [22,23].

### Plants

Air-dried leaves of *L. speciosa* were purchased from a local Giant Crape-myrtle farm in the Philippines. A certified botanical taxonomist was used to identify the plant. A voucher specimen (LS-1 L) was deposited in the Research Institute of Agriculture and Life Sciences, College of Agriculture and Life Sciences, Seoul National University.

### Bioassay-guided fractionation and isolation

Air-dried leaves (2 kg) of *L. speciosa* were pulverized, extracted with methanol (2 × 10 L) at room temperature for 2 days, and filtered. The combined filtrate was concentrated to dryness by rotary evaporation at 40°C to yield 112 g of a dark greenish powder. The extract (100 g) was sequentially partitioned into hexane- (9.35 g), ethyl acetate- (14.4 g), butanol- (39.35 g), and water-soluble (36.9 g) portions for subsequent bioassay. The organic solvent-soluble portions were concentrated under vacuum at 40°C and water-soluble portion was concentrated at 50°C. For isolation of active principles, viral CPE inhibition assay described previously [22,23] toward HRV-4 in HeLa cell was used.

**Figure 1:**
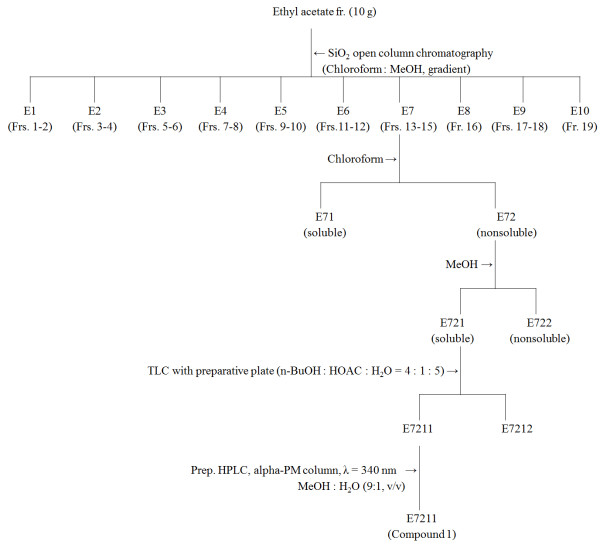
**Isolation procedures of anti-HRV principle.** The *Lagerstroemia speciosa* leaf methanol extract was sequentially partitioned into hexane-, ethyl acetate-, butanol-, and water-soluble portions. For isolation of active principles from the ethyl acetate-soluble fraction, viral cytopathic inhibition assay toward HRV-4 in HeLa cell was used.

**Figure 2:**
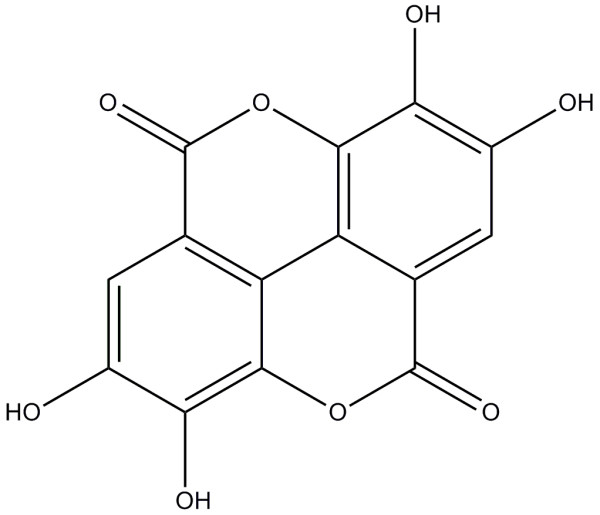
**Structure of ellagic acid.** The chemical formula is C_14_H_6_O_8_; the molecular weight is 302.197 g/mol.

The ethyl acetate-soluble fraction (10 g) was most biologically active (Table 1) and was chromatographed on a 70 × 5.5 cm silica gel (600 g) column by elution with a gradient of chloroform and methanol (100:0 (2 L), 99:1 (1 L), 95:5 (1 L), 90:10 (1 L), 80:20 (1 L), 70:30 (1 L), 60:40 (1 L), 50:50 (1 L), and 0:100 (2 L) by volume) to provide 19 fractions (each about 500 mL) (Figure 1). Column fractions were monitored by TLC on silica gel plates developed with chloroform and methanol (9:1 by volume) mobile phase. Fractions with similar *R*_f_ values on the TLC plates were pooled. Spots were detected by spraying with 10% sulfuric acid and then heating on a hot plate. Active fractions 13 to 15 (1.23 g) were pooled and separated into chloroform-soluble (474 mg) and -nonsoluble (756 mg) fractions. The active nonsoluble fraction was separated into methanol-soluble (330 mg) and -nonsoluble (426 mg) fractions. The active methanol-soluble fraction was separated by TLC plate developed with *n*-BuOH/HOAc/H_2_O (4:1:5 by volume) to give an active fraction (45 mg, *R*_f_ = 0.12). For further separation of the constituents from the active fraction, a high-performance liquid chromatography was performed. The column was a 4 mm i.d. × 200 mm EC 200/4 Nucleodex alpha-PM (Macherey-Nagel, Easton, PA) using a mobile phase of methanol and water (9:1 by volume) at a flow rate of 1 mL/min. Chromatographic separations were monitored using a UV detector at 340 nm. Finally, an active principle (35 mg) was isolated at a retention time of 2.61 min. The isolate was obtained as green amorphous powder and identified by instrumental analyses, including MS and NMR. The mass spectrum exhibited a molecular ion at *m/z* 302 [M]^+^ and IR absorption at 3380, 1720, and 1690–1610 cm^-1^ indicates the presence of phenolic hydroxyl, α-pyrone C = O, and benzonoid C = C groups, respectively. This compound (1) was thus identified as ellagic acid (2,3,7,8-tetrahydroxy-chromeno[5,4,3-cde] chromene-5,10-dione) (Figure 2). The interpretations of proton and carbon signals were largely consistent with those of Nawwar and Souleman [24]. Ellagic acid was identified on the basis of the following evidence: green amorphous power. UV (MeOH) λ_max_ nm: 255, 360. FT-IR: *ν*_max_ cm^-1^: 3380, 1720, 1690, 1610. EI-MS (70 eV) *m/z* (% relative intensity): 302 [M]^+^ (12), 278 (37), 256 (41), 105 (64), 57 (100). ^1^H NMR (DMSO-*d*_6_, 400 MHz): δ 7.47 (2H, s, ArH). ^13^C NMR (DMSO-*d*_6_, 100 MHz): δ 107.7, 110.2, 112.2, 136.2, 139.5, 147.9, 159.0.

**Table 1:** **Cytotoxicity and antiviral activity of fractions obtained from the solvent partitionings of the methanol extract of *L. speciosa *** **leaves toward human rhinovirus-4 in HeLa cells using sulforhodamine B bioassay**

**Test material**	**CC**_**50 **_**(μg/mL)**	**IC**_**50 **_**(μg/mL) (±SD)**	**TI**^**a**^
Methanol extract	1036	82 ± 1.8^d^	12.6
Hexane-soluble fraction	1074	777 ± 5.8^b^	1.4
Ethyl acetate-soluble fraction	1675	71 ± 1.0^d^	23.5
Butanol-soluble fraction	1260	881 ± 8.7^a^	1.4
Water-soluble fraction	1409	607 ± 3.5^c^	2.3

Means within a column followed by the same letter are not significantly different (*P* = 0.05, Bonferroni method).

^a^Therapeutic index = CC_50_/IC_50_.

### Antiviral assay

The antiviral activity of *L. speciosa* leaf-derived materials toward three HRVs tested was evaluated by the SRB method using CPE reduction [22,23]. In brief, HeLa cells were seeded onto a 96-well microtiter plate at a concentration of 3 × 10^4^ cells per well for 1 day. The culture medium was then removed, and the plates were washed with 1 × phosphate-buffered saline (PBS) (pH 7.3). Subsequently, 90 μL of diluted virus suspension containing CCID_50_ of the virus stock was added to produce an appropriate CPE within 2 days after infection, followed by the addition of 10 μL of MEM supplemented with 30 mM MgCl_2_ containing four to five concentrations of each test material in 0.1% dimethylsulfoxide (DMSO), based on the preliminary test results. The culture plates were incubated at 37°C and 5% CO_2_ for 2 days. After washing with 1 × PBS, 100 μL of 70% cold acetone were added to each well and left for 30 min at -20°C. After acetone solution was removed, the plates were left in a dry oven. A volume of 100 μL of 0.057% (w/v) SRB in 1% acetic acid solution was added to each well and left at room temperature for 30 min. Unbound SRB was removed and the plates were washed five times with 1% acetic acid before drying and were then left in a dry oven for 1 day. Bound SRB was solubilized with 100 μL of 10 mM unbuffered Tris-base solution and plates were left on a table for 30 min. Absorbance was read at 562 nm by using a VersaMax microplate reader (Molecular Devices, Sunnyvale, CA) with a reference absorbance at 620 nm. Ribavirin served as a positive control and was similarly prepared. Negative controls consisted of the DMSO solution. Viral inhibition rate (%) was calculated using the following formula [25]: (*OD*_tV_ – *OD*_cV_)/(*OD*_cd_ – *OD*_cV_) × 100, where *OD*_tV_ is the optical density measured with a given concentration of the test material in HRV infected cells; *OD*_cV_ is the optical density measured for the control untreated HRV infected cells; *OD*_cd_ is the optical density measured for the control untreated HRV uninfected cells.

### Cytotoxicity assay

HeLa cells were seeded onto a 96-well microtiter plate as stated previously. The culture medium was removed and the plates were replaced with media containing different concentrations of the test materials in DMSO, based on the preliminary test results. After incubation at 37°C with 5% CO_2_ for 2 days, the cytotoxicity of the test materials to HeLa cells was evaluated using a SRB assay [22,23] as stated previously.

### Infectivity of HRV particles

The effects of ellagic acid and ribavirin on the infectivity of HRV-4 particles were evaluated as described previously by Choi et al. [23]. Approximately twofold quantities of the IC_50_ values of each test compound were applied. HRV-4 was preincubated with 50 μg/mL ellagic acid or 100 μg/mL ribavirin for 1 h at 4°C. Monolayers of HeLa cells were infected with the pretreated or untreated HRV-4 for 1 h at 37°C. Unbound virus was removed by washing the wells with 2 × PBS, and then cells were incubated in fresh medium supplemented with or without test compound at 37°C. After 2 days, SRB test and antiviral activity were carried out as stated previously.

### Time course

The time-of-addition effects of ellagic acid were tested according to the method of Choi et al. [23]. In brief, monolayers of HeLa cells were seeded onto a 96-well microtiter plate as stated previously. After washing with 1 × PBS, 50 μg/mL ellagic acid were added onto the culture cells at before (-1 h), during (0 h), or after (1, 2, 4, and 6 h) HRV-4 infection at 37°C. Ribavirin served as a positive control and was similarly prepared. After 2 days, SRB test and antiviral activity were carried out as stated previously.

### Reverse transcription-PCR analysis

To evaluate the level of gene expression, quantitative real-time reverse transcription-PCR (RT-PCR) with SYBR Green dye was performed. HRV-4 infected and noninfected cultures of HeLa cells grown in Corning 25 cm^2^ cell culture flasks (Corning, NY) were treated with 50 μg/mL ellagic acid or 100 μg/mL ribavirin. After incubation at 37°C and 5% CO_2_ for 2 days, total RNA was extracted from the culture cells using the RNeasy Plus Mini Kit (Qiagen, Hilden, Germany) according to the manufacturer’s instructions. Contaminated genomic DNA was removed using RQ1 RNase-free DNase (Promega, Madison, WI). Complementary DNA was synthesized using 1 μg total RNA through a reverse transcription reaction using the SuperScript First-Strand Synthesis Kit (Invitrogen, Carlsbad, CA). Quantitative RT-PCR was performed in 96-well plates using the StepOnePlus Real-Time PCR System (Applied Biosystems, Foster, CA). Each reaction mixture consisted of 10 μL of Maxima SYBR Green/ROX qPCR Master Mix (2×) (Thermo Scientific, Foster, CA), 2 μL of forward and reverse primers (5 pmol each), 1 μL of complementary DNA (8 ng), and 7 μL of double-distilled water in a final volume of 20 μL. Oligonucleotide PCR primers for β2-microglobulin (assay ID AF072097) and HRV-4 (assay ID DQ473490.1) were purchased from Applied Biosystems. The PCR conditions were as follows: 50°C for 2 min, 95°C for 10 min, and then 50 cycles of 95°C for 15 s and 60°C for 30 s. mRNA expression level of target gene was normalized to mRNA expression level for the housekeeping gene β2-microglobulin and analyzed by the 2^–ΔΔ*C*T^ method using StepOne Software v2.1 and DataAssist Software (Applied Biosystems).

### Data analysis

Cytotoxicity was expressed as 50% cytotoxic concentration (CC_50_) of the compound that reduced the viability of cells to 50% of the control. Fifty percent inhibitory concentration (IC_50_) was defined as the compound concentration required to reducing the viral CPE to 50% of the control. The CC_50_ and IC_50_ values were calculated using GraphPad Prism 5 software (GraphPad Software, La Jolla, CA). Therapeutic index was determined as the ratio of CC_50_ to IC_50_. Results were expressed as mean ± SD of triplicate samples of three independent experiments. Statistical analyses were carried out using SAS 9.13 program (SAS Institute, Cary, NC). Data from two groups were analyzed by a Student’s *t*-test, and multiple groups were analyzed by a one-way analysis of variance and Bonferroni multiple comparison test.

## Results

### Anti-HRV activity of test compounds

The antiviral activities of natural ellagic acid, commercial pure ellagic acid, and ribavirin were significantly different from each other toward HRV-2 (*F* = 72.17; df = 2, 6; *P* < 0.0001), HRV-3 (*F* = 134.26; df = 2, 6; *P* < 0.0001), and HRV-4 (*F* = 81.32; df = 2, 6; *P* < 0.0001) (Table 2). Based on IC_50_ values, the natural ellagic acid and pure ellagic acid did not differ significantly in the antiviral activity toward three HRVs, indicating that the activity of the methanol-extracted ellagic acid is purely due to ellagic acid. Natural ellagic acid was 1.8, 2.3, and 2.2 times more toxic toward HRV-2 (IC_50_, 38 μg/mL), HRV-3 (31 μg/mL), and HRV-4 (29 μg/mL) than ribavirin, respectively. CC_50_ of ellagic acid and ribavirin was >100 μg/mL toward HeLa cells in a SRB assay.

**Table 2:** Cytotoxicity and antiviral activity of ellagic acid and antiviral agent ribavirin toward human rhinovirus-4 in HeLa cells using sulforhodamine B bioassay

**Compound**		**HRV-2**		**HRV-3**		**HRV-4**	
	**CC**_**50 **_**(μg/mL)**	**IC**_**50 **_**(μg/mL) (±SD)**	**TI**^**a**^	**IC**_**50 **_**(μg/mL) (±SD)**	**TI**^**a**^	**IC**_**50 **_**(μg/mL) (±SD)**	**TI**^**a**^
Natural EA^b^	>100	38 ± 3.2^b^	>2.6	31 ± 5.2^b^	>3.2	29 ± 2.5^b^	>3.4
Pure EA	>100	41 ± 1.1^b^	>2.4	30 ± 2.4^b^	>3.3	29 ± 1.7^b^	>3.4
Ribavirin	>100	70 ± 4.9^a^	>1.4	71 ± 0.5^a^	>1.4	63 ± 5.6^a^	>1.6

Means within a column followed by the same letter are not significantly different (*P* = 0.05, Bonferroni method).

^a^Therapeutic index = CC_50_/IC_50_.

^b^Ellagic acid.

### Effect on the infectivity of HRV particles

**Figure 3:**
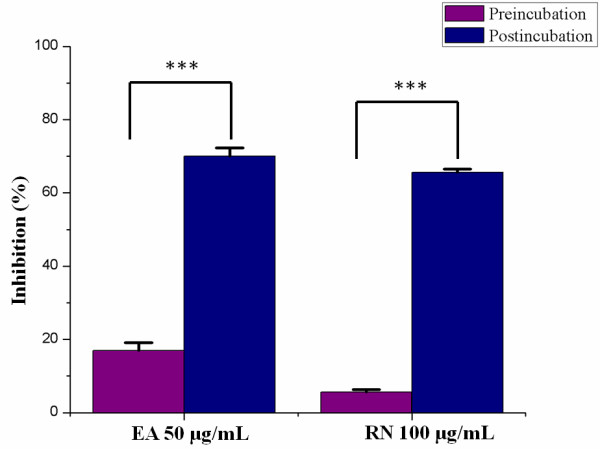
**Effect on the infectivity of HRV-4 particles.** Approximately twofold quantities of the IC_50_ values of ellagic acid (EA) and ribavirin (RN) were applied. Human rhinovirus-4 (HRV-4) particles were incubated with 50 μg/mL EA and 100 μg/mL RN for 1 h at 4°C. Afterwards, HeLa cells were incubated with treated or untreated virus for 1 h at 37°C. Unbound virus was removed by washing the wells, and infection was continued by cultivating cells in fresh medium with or without test compound at 37°C. After 2 days, inhibition was evaluated by SRB method and expressed as the inhibition rate. Each bar represents the mean ± SD of triplicate samples of three independent experiments. ^***^Significant at *P* < 0.001, according to a Student’s *t*-test.

The effects of ellagic acid and ribavirin on the infectivity of HRV-4 particles were likewise examined (Figure 3). The inhibition rates of preincubation with 50 μg/mL ellagic acid and 100 μg/mL ribavirin were 17 and 5.7%, respectively. Continuous presence of ellagic acid and ribavirin during infection led to a significant increase in the inhibition rate (70 and 65.7%).

### Time course of compound addition

**Figure 4:**
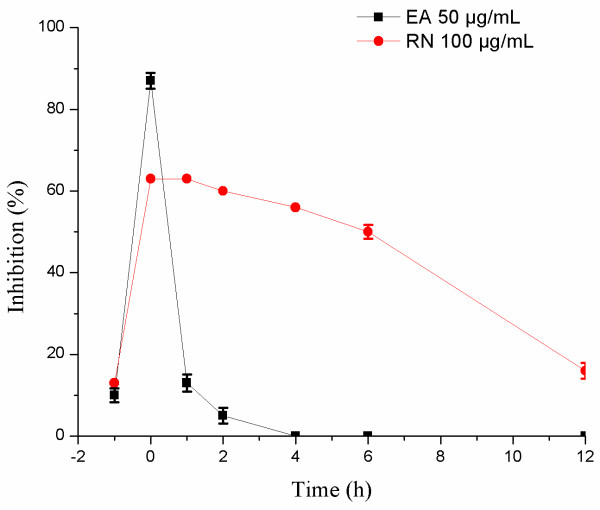
**Time-of-addition effect on HRV-4 replication in HeLa cells.** Approximately twofold quantities of the IC_50_ values of ellagic acid (EA) and ribavirin (RN) were applied. The 50 μg/mL EA and 100 μg/mL RN were added at various times preinfection (-1 h), coinfection (0 h), or postinfection (1, 2, 4, and 6 h) of human rhinovirus-4 (HRV-4) to HeLa cells at 37°C. After 2 days, inhibition was evaluated by SRB method and expressed as the inhibition rate. Each bar represents the mean ± SD of triplicate samples of three independent experiments.

To investigate the mode of action of ellagic acid and ribavirin, time course of the inhibition of these compounds at different periods (before, during, and after) of HRV-4 infection was likewise investigated (Figure 4). Treatment with 50 μg/mL ellagic acid considerably suppressed HRV infection only when added just after the virus inoculation (0 h) (87% inhibition). The inhibition rate of ellagic acid declined to 20% or less when added at either prior (-1 h) or post (1, 2, 4, and 6 h) infection. However, the inhibitory effect of 100 μg/mL ribavirin on HRV-4 infection occurred between 0 and 6 h.

### Effect on the level of HRV replication

**Figure 5:**
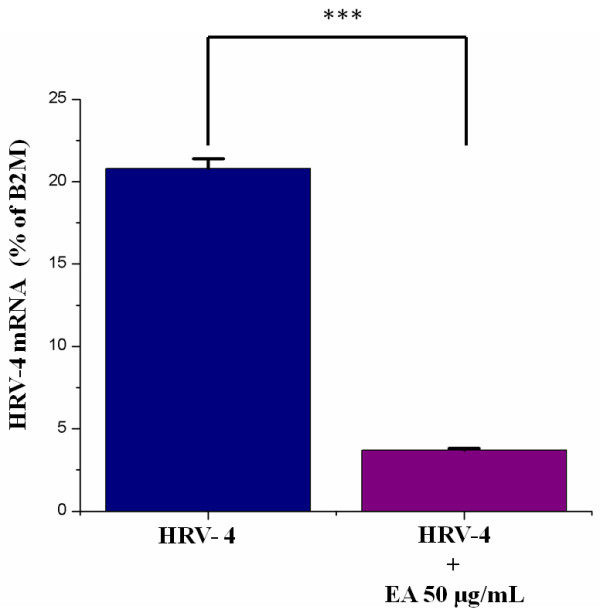
**Effect on replication level of HRV-4.** The RNA replication level of human rhinovirus (HRV-4) was detected by real-time reverse transcription-PCR with SYBR Green dye in HeLa cells 2 days after infection in the presence of 50 μg/mL ellagic acid (EA). HRV RNA expressions were normalized to the constitutive expression of mRNA of the housekeeping gene β2-microglobulin (B2M) and analyzed by the 2^–∆∆*C*T^ method using StepOne Software v2.1 and DataAssist Software. Each bar represents the mean ± SD of duplicate samples of three independent experiments. ^***^Significant at *P* < 0.001, according to a Student’s *t*-test.

The RNA replication level of HRV-4 in HeLa cells was remarkably inhibited in the cell cultures treated with 50 μg/mL ellagic acid (Figure 5). The RNA replication level of HRV-4 was reduced by 5 fold, compared to the level in the cell cultures without ellagic acid.

## Discussion

The current *in vitro* study indicates that materials derived from *L. speciosa* leaves exhibited antiviral activity and selectivity toward three rhinoviruses HRV-2, HRV-3, and HRV-4. The plant grows widely in tropical countries, including the Philippines, India, Malaysia, China, and Australia and is a popular folk medicine in Southeast Asia. *L. speciosa* leaves contain acetal, alkaloids, sterols, tannins, and triterpenoids [26,27]. Excellent antidiabetic properties of a standardized extract from *L. speciosa* leaves have been well noted [28,29].

Various compounds, including phenolics, terpenoids, and alkaloids, exist in plants, and jointly or independently they contribute to antiviral efficacy [14]. Many plants and their constituents manifest antiviral activity toward different viruses [14,30] and have been proposed as alternatives to conventional antiviral drugs. Anti-HRV constituents derived from plants include alkaloids (e.g., arborinine and (*S*)-ribalinine, IC_50_ 3.19 and 82.95 μM [31]; glaucine, IC_50_ 22 μM [32]), coumarins (e.g., 6,7,8-trimethoxycoumarin and daphnoretin methyl ether, IC_50_ 11.98 and 97.08 μM [31]; farnesiferol B and C, IC_50_ ≈ 2.61 μM [33]), flavonoids (e.g., 4′,5-dihydroxy-3′,3,7-trimethoxyflavone, IC_50_ 0.29 μM [34]; 3-methylquercetin and three related compounds, effective concentration 15.8 μM [35]; chrysosplenol D and others, minimum effective dose 0.22–33.3 μM) [36]; chrysosplenol C, IC_50_ 0.75 μM [37]), terpenoids (e.g., 3-*O*-*trans*-caffeoyltormentic acid, IC_50_ 30.72 μM [38]; orobol 7-*O*-D-glucoside, IC_50_ 1.29–19.62 μM [39]), organic acid (e.g., raoulic acid, IC_50_ 0.51 μM [40]; gallic acid, IC_50_ ≈ 294.55 μM [41]), and thiosulfinates (allicin and allyl methylthiosulfinate [42]). It has been reported that HRV capsid-binding compounds toward all HRV serotypes showed the existence of group A and B, based on a wide range of susceptibilities to antiviral compounds [43]. In the current study, the antiviral principle was determined to be the tannin ellagic acid. The constituent exhibited antiviral activity toward both group A (HRV-2) and group B (HRV-3 and HRV-4). IC_50_ of ellagic acid was between 95.9 and 125.8 μM toward three HRVs, although IC_50_ of the natural compounds stated previously is between 0.22 and 294.55 μM. Ellagic acid exhibited greater antiviral activity than ribavirin toward three HRVs and high selectivity. This original finding indicates that materials derived from *L. speciosa* leaves can hold promise for the development of novel and effective naturally occurring antiviral agents for two different HRV groups (A and B). In addition, ellagic acid was reported to possess anti-HIV activity, through inhibition of HIV protease [44]. Orobol 7-*O*-D-glucoside from *L. speciosa* leaves is also known to have broad-spectrum antiviral activity toward various HRVs including group A and B, as well as pleconaril-resistant HRV-5 [39].

Investigations on the modes of antiviral action of naturally occurring compounds may contribute to the development of selective HRV therapeutic alternatives with novel target sites. The modes of anti-HRV action of plant secondary substances have been well reviewed by Rollinger and Schmidtke [45]. Targeting virus molecules is likely more specific and less toxic, but there is a narrow spectrum of viruses and a higher risk of creating resistant viruses [46]. On the contrary, chemicals which target cellular molecules may possess a broader antiviral activity spectrum and less risk of developing virus resistance, but may be more toxic to the host cell [46]. In the current study, ellagic acid does not interact with the HRV-4 particles, as preexposure of the virus to the constituent did not alter the infectivity of HRV-4 particles. Based on time-of-addition experiments, ellagic acid significantly suppressed HRV-4 infection only when added just after the virus inoculation (0 h), but not before -1 h or after 1 h or later. This finding suggests that ellagic acid may directly interact with the human cells in the early stage of HRV infections to protect the cells from the virus destruction. In addition, RT-PCR analysis revealed that ellagic acid strongly inhibited the RNA replication of HRV-4 in HeLa system, suggesting that ellagic acid inhibit virus replication by targeting on cellular molecules, rather than virus molecules. Detailed tests are needed to fully understand the anti-HRV mode of action of ellagic acid.

## Conclusions

*L. speciosa* leaf-derived preparations containing ellagic acid could be useful as an antiviral agent in the prevention or treatment of HRV infection. The antiviral action of ellagic acid may be an indication of at least one of the pharmacological actions of *L. speciosa*. For the practical use of *L. speciosa* leaf-derived preparations as novel anti-HRV products to proceed, further research is needed to establish their human safety and whether this activity is exerted *in vivo* after consumption of *L. speciosa* leaf-derived products by humans. Historically, a tea from the plant leaves has been used for the treatment of diabetes mellitus in the Philippines [28]. Rats fed ellagic acid at doses as high as 50 mg/day up to 45 days did not cause any signs of systemic toxicity [47]. Lastly, detailed tests are needed to understand how to improve anti-HRV potency and stability for eventual commercial development.

## Abbreviations

ATCC: American type culture collection; CC_50_: 50% cytotoxic concentration; CCID_50_: 50% cell culture infective dose; CPE: Cytopathic effect; DMSO: Dimethylsulfoxide; HRV: Human rhinovirus; IC_50_: 50% inhibitory concentration; MEM: Minimum essential medium; PBS: Phosphate-buffered saline; RT-PCR: Reverse transcription-PCR; SRB: Sulforhodamine B; TLC: Thin layer chromatography.

## Competing interests

The authors declare that they have no competing interests.

## Authors’ contributions

YJA conceived the project and supervised the study. SWP, MJK, and JYY designed the experimental protocol, carried out the tests, analyzed data, and wrote the first draft of the manuscript. HJC helped to develop the protocol and analyze the data. YJA evaluated the results and wrote the final draft of this manuscript. All of the authors read and approved the final manuscript.

## Pre-publication history

The pre-publication history for this paper can be accessed here:

http://www.biomedcentral.com/1472-6882/14/171/prepub
